# Membrane Trafficking and Subcellular Drug Targeting Pathways

**DOI:** 10.3389/fphar.2020.00629

**Published:** 2020-05-27

**Authors:** Ajay Kumar, Anas Ahmad, Akshay Vyawahare, Rehan Khan

**Affiliations:** Department of Nano-Therapeutics, Institute of Nano Science and Technology, Mohali, India

**Keywords:** membrane trafficking, drug targeting pathways, membrane vesicle (MV), nanocarrier and delivery, subcellular transport

## Abstract

The movement of micro and macro molecules into and within a cell significantly governs several of their pharmacokinetic and pharmacodynamic parameters, thus regulating the cellular response to exogenous and endogenous stimuli. Trafficking of various pharmacological agents and other bioactive molecules throughout and within the cell is necessary for the fidelity of the cells but has been poorly investigated. Novel strategies against cancer and microbial infections need a deeper understanding of membrane as well as subcellular trafficking pathways and essentially regulate several aspects of the initiation and spread of anti-microbial and anti-cancer drug resistance. Furthermore, in order to avail the maximum possible bioavailability and therapeutic efficacy and to restrict the unwanted toxicity of pharmacological bioactives, these sometimes need to be functionalized with targeting ligands to regulate the subcellular trafficking and to enhance the localization. In the recent past the scenario drug targeting has primarily focused on targeting tissue components and cell vicinities, however, it is the membranous and subcellular trafficking system that directs the molecules to plausible locations. The effectiveness of the delivery platforms largely depends on their physicochemical nature, intracellular barriers, and biodistribution of the drugs, pharmacokinetics and pharmacodynamic paradigms. Most subcellular organelles possess some peculiar characteristics by which membranous and subcellular targeting can be manipulated, such as negative transmembrane potential in mitochondria, intraluminal delta pH in a lysosome, and many others. Many specialized methods, which positively promote the subcellular targeting and restrict the off-targeting of the bioactive molecules, exist. Recent advancements in designing the carrier molecules enable the handling of membrane trafficking to facilitate the delivery of active compounds to subcellular localizations. This review aims to cover membrane trafficking pathways which promote the delivery of the active molecule in to the subcellular locations, the associated pathways of the subcellular drug delivery system, and the role of the carrier system in drug delivery techniques.

## Introduction

**Figure 1:**
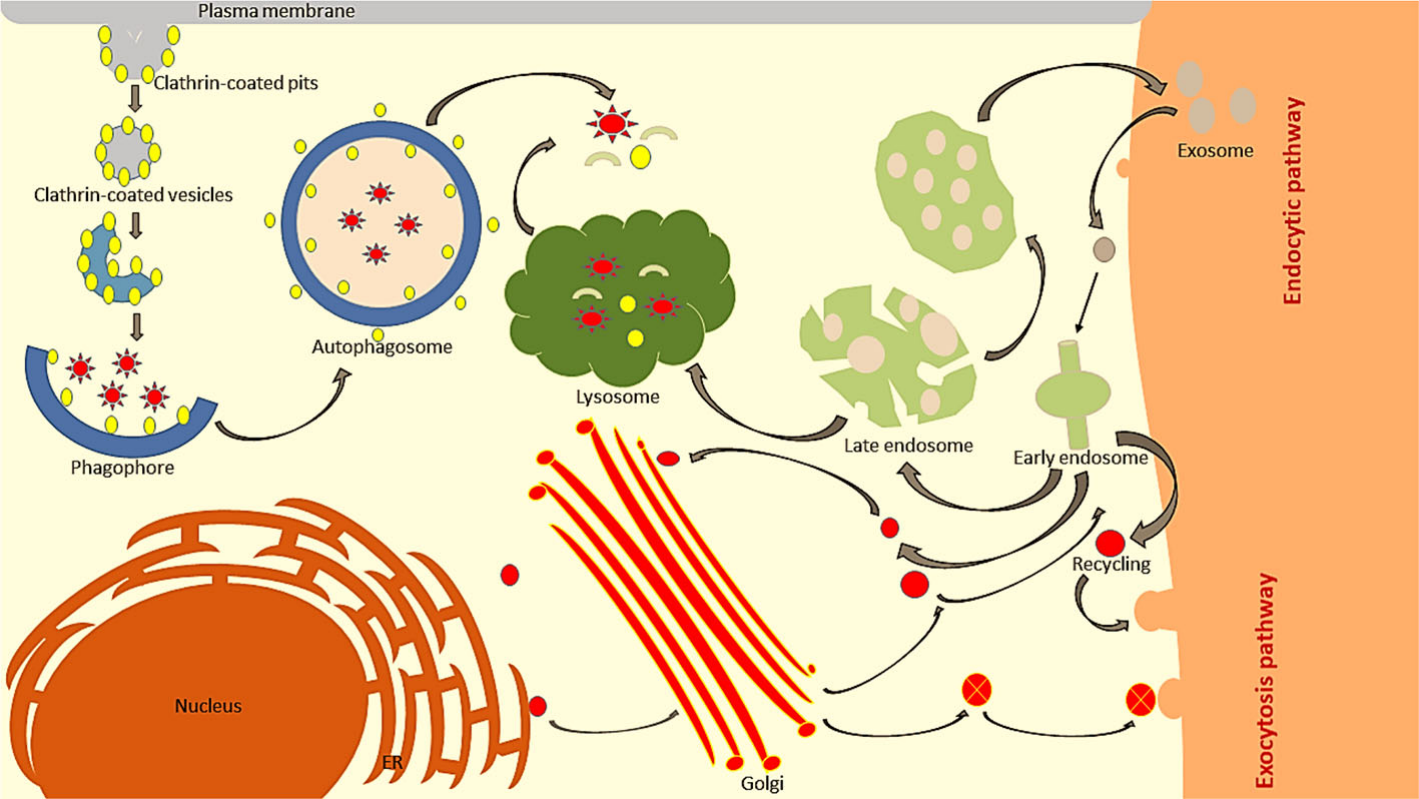
In eukaryotic cells, membrane trafficking is divided into two major pathways. In endocytosis, proteins are internalized from the outer cell surface into the early endosomes. The early endosome determines the trafficking route for the internalized material, either by sending it to the plasma membrane for recycling, degradation *via* the lysosome, or for retrieval by the trans-Golgi network. On the other hand, exocytosis translocates newly synthesized proteins into the ER, entering further into the cis-Golgi complex and is transported *via* the trans-Golgi network.

For a cell to live, it needs a constant flow of nutrients and the removal of unwanted or used materials. Formation, fusion, and trafficking of membranous vesicles are processes that commonly occur within the cell, which regulate the fate of cells (Tokarev et al., 2013). These processes control vital cellular activities such as: cellular uptake, transportation of nutrients for metabolism and pathogens for degradation, trafficking of moieties to the desired location, transportation of signalling molecules, communication within or outside cells, and many others (Muro, 2018). In order to maintain the transportation of proteins, lipids, and solutes, there are specialized pathways that work within the cells called membrane trafficking pathways. Membrane trafficking is a key process for maintaining the sustainability of the cell by transporting these nutrients and other solutes to all parts of the cellular system (Ebine and Ueda, 2015). Membrane trafficking is divided into two basic movement pathways viz. endocytosis and exocytosis. Their function is to control the movement of cargo and drug payloads to the plasma membrane or out of the cell. These specialized pathways also deliver the newly synthesized proteins to their desired locations inside the cells (**Figure 1**) (Watson et al., 2005).

These trafficking phenomena exist both at the cell membrane as well as subcellular levels and have been exploited directly, or otherwise, to regulate the localization and placement of both endogenous and exogenous molecules. The attributes of the cell membrane, which govern the membrane trafficking, include, but is not limited to membrane potential, its fluidity, and alterations in its competency (Xue et al., 2013; Staudt et al., 2016). Drug resistance develops frequently as a result of altered membrane trafficking, which culminates into reduced drug uptake by membranous transporters localized at the cell surface (Xue et al., 2013). In other instances, subcellular trafficking mechanism, organelle function, and other complex molecular interplays are manipulated to achieve the maximum potency of drugs and other agents (Striebinger et al., 2015). The major characteristics which regulate subcellular trafficking include modification in the subcellular pH and modification in the cell cycle parameters. (Abdrabou and Wang, 2018) Furthermore, membrane trafficking is a complicated channelization phenomena composed of multiple trafficking vesicles and some extra vesicular networks that are involved in endocytosis, exocytosis, as well as cellular autophagy. These processes necessitate the aid of several subcellular transportation components like early endosomes, late endosomes, lysosomal networks etc (Zhang et al., 2016).

Strategies are being employed to design formulations that act as prodrugs, bearing the components that closely resemble the cell membrane constituents so as to trace and monitor their cellular handling viz. membrane and subcellular trafficking events like fusion, translocation, internalization, carrier sequestration, perinuclear localizations, drug release etc (Maji et al., 2018). The selective delivery of therapeutic molecules to specific diseased sites also involves these membranous and subcellular trafficking components. For example, in the case of cancer therapy, delivering a therapeutic payload to the vicinity of the nucleus demands not only the preferential recognition of cancerous cells and the nucleus but also the payload entry and its subsequent transportation *via* membranous and subcellular trafficking. This will impart selective damage to the targeted cancer cells by delivering the drug in its extreme vicinity while causing minimal harm to the adjacent normal cells and tissues (Rosenkranz et al., 2019). It is only the membrane trafficking, by which cellular and subcellular transport of micro and macromolecules takes place, that substantially governs the essential cellular functions and responses like drug interaction with plasmid DNA, transfection efficiencies, cellular uptake capabilities, internalization mechanisms, cellular and subcellular localizations, and other pharmacokinetic and pharmacodynamic paradigms of the therapeutic molecules (Mazzaglia et al., 2018).

Targeting the membrane trafficking of enzymes involved in the disease pathology is another perspective which exploits the subcellular localization mechanism. Rajendran et al., described a protocol which targeted the membrane bound β-secretase transition state by an inhibitor rather than targeting the active binding site of the enzyme. This membrane targeting involves endosomes and enhances the local membrane density of inhibitor and imparts more enzyme inhibitory efficiency both *in vitro* and *in vivo*. The authors suggested that this scheme can significantly benefit the formulation of potent therapeutic molecules against membrane protein targets (Rajendran et al., 2008).

Another aspect of membrane trafficking constitutes the design of targeting strategies such that some very specific molecules viz. folate receptors (Slastnikova et al., 2017), epidermal growth factor receptros (Rosenkranz et al., 2018), melanocortin receptor-1 (Durymanov et al., 2012), and α-melanocyte stimulating hormone (Rosenkranz et al., 2003), overexpressed on the diseased (e.g. cancerous) cells, act as the target and receptor-specific ligand bearing transportation carriers that are designed for the delivery of therapeutic payloads. The carriers aid in the receptor binding, internalization, endosomal escape, and nuclear internalization. As the nucleus is the most sensitive and vulnerable subcellular locality, the translocation domain of the carrier helps the drug disposition in the vicinity of nuclear DNA of the receptor overexpressing cells and significantly enhances the cancer cell lethality as a result of the increased therapeutic efficacy of drugs (Rosenkranz et al., 2003; Durymanov et al., 2012; Slastnikova et al., 2017; Rosenkranz et al., 2018).

Although a number of reviews have previously been published, these reviews have mainly been restricted in their focus on either only subcellular drug targeting or subcellular drug targeting with respect to drug delivery. The study by Harisa and Faris (2019) mainly emphasizes the limitations of drug targeting into intracellular compartments and factors affecting molecular entry into the compartment. Likewise, Liu et al. (2020)  focus on the limitations of traditional nanocarriers by explaining their pathways and principles with respect to drug targeting. The emphasis is made on challenges which arise in cancer therapy by the nanocarriers. Similarly, Nag and Delehanty (2019) discussed drug delivery with respect to cellular and subcellular targeting. The review mainly focused on recent advancements in nanoparticle mediated drug delivery techniques. Donahue et al. (2019) discussed the physiochemical properties of nanoparticles affecting cellular internalization and cellular interaction. As the movement of molecules within the cell is vitally governed by the complex process of membrane trafficking, and in order to achieve subcellular drug targeting, an in-depth understanding of membrane trafficking is of utmost significance. The present review therefore focuses, in detail, on the role of membrane trafficking in subcellular drug targeting along with various trafficking mechanisms and the carrier mediated targeting of cellular and subcellular trafficking pathways.

## Membrane Trafficking

### Overview

The complexity of mammalian cells is accompanied by the compartmentalization and flow of newly synthesized proteins, nutrients, and molecules and is maintained by the inbuilt trafficking system. There are a number of pathways that control the movements, like the biosynthetic pathway for proteins, or the endocytic pathway for molecules such as ligands and solutes entering cells (Watson et al., 2005). Membrane trafficking provides the fundamental needs of cells to maintain homeostasis and creates a flow of materials for the signalling process. The eukaryotic system of membrane trafficking started from endoplasmic reticulum, followed by Golgi apparatus, endosomes and lysosomes (Cheung and de Vries, 2008).

When the drug binds to its receptors, the internalization of the ligand takes place through endocytosis. One of the main steps that involves the intracellular delivery of molecules includes the formation of vesicles which squeeze out from the plasma membrane to deliver intracellularly. Mainly through the activity of GTPase, dynamin is required for this step and it is therefore the mechanochemical enzyme that initiates the formation and membrane fission of vesicles. According to another model GTPases hydrolysis dismisses the communication with the effector (Vincent, 2003).

The major pathways governing the trafficking of molecules such as the receptor, ligands, and solutes are known as endocytic pathways which control the entry into cells. The trafficking of pharmacological agents *via* endocytotic pathways requires intracellular membrane trafficking and molecular dynamics of the concerned organelles. This flux is maintained by the individual compartments and endocytic pathways. The trafficking of proteins and lipids is maintained by balanced targeting, retention, and retrieval mechanisms. The most effective site for delivering drugs to the targeted location is the endocytotic pathway. Nowadays, targeting of drugs to the specific location requires actively checking the exclusivity of the desired action either within the organelles or it requires the delocalization of endosomes or lysosomes prior to the targeted location. Drug release therefore depends on the early endosomal escape as compared to the utilization of lysosomal protease mediated drug release (Watson et al., 2005).

### Components of Membrane Trafficking

Intracellular membrane transport systems responsible for trafficking nearly all the molecules are expressed in mammalian cells. To perform this orchestra in a pristine manner, some major components such as: endoplasmic reticulum, Golgi apparatus, endolysosomal platform, and a molecular machinery to hold approximately 2,000 proteins, actively partakes. It begins with endoplasmic reticulum, a complex system consisting of tubules and cisternae. It prepares the protein for its journey by glycosylation and folding. Sometimes it unfolds the folded protein in certain stress periods and governs the fate of the cell. After the complex folding step is completed the protein is ready to move to its next location by exiting the endoplasmic reticulum as vesicles, which are either small or large, with the help of the coat protein complex II (COPII). With the help of COPI, the cargo reaches the Golgi complex and takes part in glycosylation from the cis part of Golgi to trans part and be packed, according to their cargo, by specialized membranous carriers like- lysosomes or uncoated pleomorphic carriers in the form of secretory granules destined to plasma membrane (De Matteis and Luini, 2011).

### Regulating Membrane Trafficking

In order to maintain homeostasis, cell needs to sense the surrounding environment and acts accordingly by communicating *via* the release of mediators and the signalling molecule. Multiple receptor mediated signalling pathways are coordinated for many important physiological processes. One of them, which belongs to the largest family of signalling receptors, is G Protein Coupled Receptors (GPCRs). The endocytic trafficking of GPCR is a result of studies related to ligand-induced desensitization of receptor-mediated signalling. There have been several studies on GPCRs, especially the β_2_-adrenergic receptor, which showed rapid desensitization *via* receptor phosphorylation. The study showed trafficking of agonist-bound receptor signalling *via* the upregulation of heterotrimeric G proteins which rapidly and selectivity undergo phosphorylation by GPCR kinases (GRKs) over the agonist-activated receptor *via* phosphorylation. Phosphorylation of the receptor and binding with β-arrestin prevents interaction between the receptor and G-protein, leading to termination of G-protein-mediated signalling. The sorting of the signalling receptor can occur at any point in the endocytic pathway. First, the plasma membrane GPCRs undergo ligand-induced concentration in clathrin-coated pits and some selective endocytosis *via* clathrin-independent mechanisms, which later affects endocytic trafficking (Hanyaloglu and von Zastrow, 2008).

For the complex regulatory mechanism of sorting, the intracellular membrane trafficking pathway is accompanied by multi-level specific components of transport including proteins such as SNAREs, Rab GTPases, and Sec1 homologs, for the maintenance of vesicle-docking and vascular-fusion reactions. The function of phosphatidylinositol and its phosphorylated derivatives, known as phosphoinositides, are primarily introduced as second messengers in signal transduction pathways. Recently, however, membrane trafficking regulated by the phosphoinositide-signalling pathway, has come under attention. The genetic study conducted for protein transport to the yeast vacuole or lysosome showed the presence of the VPS34 gene encoded for PI 3-kinase, involved in the phosphorylation of PTDIns at the n-3 position of the inositol ring, for the production of PtdIns3P. Vps34p is activated by the VPS15 gene product and acts as a membrane-associated serine/threonine protein kinase which catalyzes an auto-phosphorylation reaction (Odorizzi et al., 2000).

## Targeted Drug Delivery Techniques and Pathways

### Overview

The human body is a complex network of different organs, tissues, cells, and different organelles, with various pathways that control the cellular activities of the human body. However, sometimes, due to some internal or external factors, the cellular environment becomes disturbed and the body tries to maintain homeostasis but is unable to do so. This results in a disease of that particular organ. To treat that particular disease condition, drugs are available to antagonize the processes and causes of the disease and to normalize the biological conditions. These drugs are administered *via* various routes to target the particular organ affected, but, due to a complex cellular network, the administered medication cannot reach the desired target if it is located at subcellular levels (Manish and Vimukta, 2011). To target subcellular components, researchers need to develop certain carriers which can encapsulate the drug inside it and protect it from various barriers such as pH, efflux pump proteins, enzymes, and also from the body's immune responses. Earlier, various drug delivery systems were developed. Very few of these drug delivery systems successfully reached the market and many failed at various levels of clinical settings (Rani and Paliwal, 2014). Targeting a particular gene, receptor, ion channel, or enzyme through a particular moiety, which is responsive to those stimuli, could be a more effective way to tackle these delivery barriers. The course of travelling of NPs into and then further inside the cell is very complex and is mainly governed by unique endocytic pathways, which mainly depend on the particular cell type and ligand (Chou et al., 2011). Transferrin (Tf), is an iron binding protein which selectively activates transferrin receptors and becomes internalized through the clathrin-mediated endocytic pathway (Foroozandeh and Aziz, 2018). Following the course of endocytosis, researchers are more inspired by targeting intracellular trafficking networks or pathways. Research is therefore nowadays focused on the development of intracellular targeted drug delivery techniques.

### Types of Drug Delivery Systems

Various membrane trafficking pathways control the cellular behaviour, and, by exploiting these pathways researchers have tried to develop novel ways to tackle problems such as drug resistance, e.g. by targeting the efflux pump inhibitor cyclosporine A encapsulated into the polymeric PEO-b-PCL micelles (Aliabadi et al., 2005; Yang et al., 2008). Mitochondria are key regulators of the energy and power house organelles inside the cells. These are involved in various biological processes such as fatty acid metabolism, Kreb's cycle, and oxidative phosphorylation. These are also involved in apoptosis by activating the cell death process through different mechanisms. Delocalized lipophilic cations (DLC's) e.g. Methyltriphenylphosphonium (Murphy, 1997) are highly lipophilic and cationic in nature and readily pass through the mitochondrial membrane, due to negative membrane potential (Chen et al., 2016). Liming Wang et al., developed serum protein coated Au-Nanorods (Au-NRs) for the treatment of cancer. They studied the effect of Au-NRs on A549 cell lines and found that Au NRs are selectively lethal for the cells. TEM analysis showed that these enter through the plasma membrane and are internalized in A549 cells and then into the endosomes, lysosomes, and mitochondria. The nanoformulations are reported to inhibit the growth of cells by increasing the ROS level in cancer cell mitochondria (Wang et al., 2011).

### Advantages of Targeted Drug Delivery Techniques

The nanocarrier system for intracellular targeting has emerged as the most preferred vehicle for drug delivery in the case of cancer treatment. There are various advantages of intracellular targeting and these include the ability to bypass the mononuclear phagocyte system (MPS), the triggered release of payloads, selective targeting, improved pharmacokinetics, longer half-life, improved bioavailability, efficient navigation, and multivalent surface modification (Rejman et al., 2004; Xu et al., 2015; Jhaveri and Torchilin, 2016). This recent approach deals with the functionalization of nanoparticles with multiple functional moieties so as to fulfill multiple purposes. The multivalent functionalization platforms perform various functions like enhancing the cellular uptake, more transfection efficiencies, and precise and effective targeting of subcellular organelles etc. (Chen et al., 2017; Peng et al., 2017; Sun et al., 2017). Sometimes the nanoparticulate platforms are used for minimization of the reaction distances between different reactants such that the reaction efficiencies can be maximized. In one such instance, novel upconversion nanoparticles (UCN) were used for a photosensitizer protoporphyrin IX (PpIX) to enhance the ROS production to gain the advantage of subcellular positioning and irradiation productivity. This platform was used for mitochondrial targeting and exhibited significantly enhanced tumor cell killing and the inhibition of tumor growth (Chen et al., 2017).

The functionalization of nanoparticles with a single ligand is often not sufficient enough to achieve the desired level of cell surface or subcellular organelle targeting which ultimately hampers the best therapeutic efficiency of the delivered drug payload. Therefore, nanoparticles conjugated with dual peptides e.g. galectin-3-targeted G3-C12 peptide and _D_(KLAKLAK)2 (KLA) peptide were employed to target the mitochondria after undergoing receptor mediated internalization into galectin-3-overexpressing PC-3 cells. This approach provides multiple advantages such as enhanced mitochondrial membrane disruption, significantly more ROS generation, and much better cytotoxic effects. It also resulted in better tumor accumulation of the delivery system and the best therapeutic efficiencies were achieved with a better animal survival rate (Sun et al., 2017). Another similar approach for particles decorated with two targeting moieties involves one of these binding to folate membrane cell surface receptors and the second (triphenylphosphine, TPP) targeting the mitochondrial membrane. This approach offers the benefits of fine control in the targeting process through asymmetric attachment of ligands on each side of the particles. The folate receptor targeting offers enhanced cellular uptake and accumulation inside tumor cells followed by mitochondrial targeting with TPP and improved the therapeutic efficacy of the nanomedicine (López et al., 2017).

Sometimes the nanoparticulate platform in itself is composed of a self-assembling or self-aggregating moiety such as triphenylphosphonium-appended coumarin and loaded with an anticancer drug, e.g. doxorubicin, to achieve the selective mitochondrial targeting. These nanocarriers show the benefits of selective mitochondrial accumulation and possess the advantage of efficient doxorubicin delivery to mitochondria. This kind of nanomedicinal platform possesses significant clinical potential in being a specific sub-organelle targeted delivery system for anti-cancer therapeutics (Lee et al., 2018). Along with these benefits, the nanoparticles targeting cellular and subcellular locations offer other distinct advantages in terms of their physicochemical, morphological, and functional properties, which are of specific interests to researchers. Further advancements in terms of their improved selectivity, maximum therapeutic outcome, precise entry into cancer cells *via* targeting ligands, and responsiveness to physical or biological stimuli help in their enhanced subcellular performances (Liu et al., 2020).

### Subcellular Drug Delivery Pathways

Successful treatment of any disease can only be achieved when the drug, molecules, proteins, si-RNAs, DNAs, *via* efficient carrier systems, are effectively administered at the subcellular level of the targeted cells. Various drug delivery techniques are followed to reach the subcellular level. One effective way to reach the subcellular level is achieved by targeting the membrane trafficking pathways (Hayes et al., 2006). The therapeutic effects will appear as a result of target binding of the specialized drug molecule at subcellular levels. The subcellular targeting system requires one to follow some of the criteria - they should not be captured by degradation pathways, capable of receptor mediated endocytosis and target recognition (**Table 1**). It should be able to release the drug at the desired site and also maintain the therapeutic concentration in the cells (Chou et al., 2011). In an attempt Durymanov et al., synthesized a new PEI-PEG based polyplexes loaded with a MC1SP-peptide which are specific for melanocortin receptor – 1 and investigated its potential. The synthesized and targeted polyplexes showed greater efficacy in receptor-mediated transfection of cloned (Cloudman S91) murine melanoma cells as compared to non-targeted polyplexes. In addition, the inhibition of transfection was mediated by chlorpromazine, a positive inhibitor of the clathrin-mediated endocytosis pathway. The synthesized polyplexes also showed rapid nuclear uptake as compared to non-targeted polyplexes (Durymanov et al., 2012).

**Table 1:** List of endocytosis pathways and targeting ligands.

Type of Endocytosis	Targeting agents	Mediated by	Mechanism of internalization	Delivery vehicle	Ref.
Phagocytosis	Folic acid	Neutrophils, macrophages	Caveolin-assisted	Ligand	(Pan et al., 2018)
Macropinocytosis	Non-specific	Ras-activated PI3-kinases (class-1 PI3-kinase), Ras and probably the protein kinase Akt (which binds PIP3)	Actin-driven endocytic pathway	–	(King and Kay, 2019)
Clathrin mediated	Mannose-6-phosphate, Transferrin, Riboflavin	Majority of cells	Clathrin-Dependent RME	Human serum albumin, PEGylated Liposomes, Polymeric chitosan vesicles, Bovine serum albumin	(Bareford and Swaan, 2007)
Caveolae-mediated	–	Cells expressing Caveolin-I or have Caveolin-3	Caveosomes *via* Caveolin protein	–	(Shajahan, )
Clathrin and Caveolin-independent	glycosylphosphatidylinositol (GPI)-anchored proteins, glycosphingolipid-binding toxins	GTPase Dynamin	Clathrin coated pits	–	(Hansen and Nichols, 2009)

The natural cell transport process was exploited for intracellular targeting of the auger electron emitter. The advantage of auger electrons, found in the decay product of many radionuclides, is the damage produced by these short range auger electrons in the local vicinity with minimal damage to adjacent normal cells, and potentiates its use for targeting cancer cells. The ability of auger emitters to localize preferentially in the cellular nuclei is exploited by researchers in cancer therapy (Rosenkranz et al., 2019). Rosenkranz et al., developed a specific transporter for cell specific nuclear delivery for locally acting drug therapy. The transporters were comprised of a melanocyte stimulating hormone, a nuclear localization sequence, and a protein carrier such that it can translocate into the nucleus and deliver the desired drug at the vulnerable site of nuclear DNA. This paradigm was able to deliver the drug to cell specific particular subcellular locations and resulted in enhanced therapeutic efficacy (Rosenkranz et al., 2003). Similar targeted therapeutic delivery platforms were exploited *via* other targeting approaches, formulations, and characterization of folate receptor targeted nanotransporters for cancer specific cell accumulation and cancer cell nuclei eradication (Slastnikova et al., 2017). In a similar fashion, EGFR over expressing bladder cancer cells were targeted for precise drug delivery in the vicinity of nuclear DNA such that high toxicity is precipitated in the cancer cells. The nanoformulation employed here significantly enhanced *in vitro* and *in vivo* therapeutic effects of the delivered molecules (Rosenkranz et al., 2018). There are various strategies that are discussed under this topic.

#### Passive Targeting

Passive drug targeting takes advantage of the biological system but does not attach any active moiety or targeting molecule in the drug delivery system. Some of these are used to target the drug molecule at the mononuclear phagocytic system, enhanced permeability, and retention effect or to target the local organ. In another way it can be the chemoembolization or the pegylation might enhance the stability and stealth effect. Hepatic chemoembolization is one of the methods used to target liver cancer or liver metastasis by injecting chemotherapeutic drug containing particles, which are specially made to halt the supply of nutrients and oxygen directly into the cancer cell arteries, and are able to inhibit the growth of cancer cells (Bruix et al., 2004). Enhanced permeability and retention effect (EPR) occurs mainly due to leakiness of the micro-vascular structure of tumor blood vessels. By administering drugs, it can therefore easily enhance the retention of the drug.

#### Active Targeting

Active targeting consists of targeting only diseased cells and rendering healthy cells unaffected. The main targets are overexpressed markers such as enzymes, proteins, receptors, and ion channels. To target these overexpressed markers researchers conjugated a drug carrier system with a specific ligand or substrate of that particular marker which mediates some ligand-receptor, enzyme-substrate interactions (Zheng et al., 2006; Gao et al., 2019). Lysosomal targeting is also an effective way of treating defective cells. Lysosomes contain a variety of hydrolases which are mainly involved in degradation, autophagy, recirculation, membrane repair, and secretions. The acidic environment (pH~5) of lysosomes makes it suitable for target therapies that control the release of drugs at acidic pH. Ligand conjugated nanoformulations majorly target the lysosomes *via* receptor mediated endocytosis (Shen et al., 2017). Mitochondria are known as the power house of cells, and researchers have therefore tried to enhance the ROS (reactive oxygen species) level, to induce mitochondrial damage, and blockage of the respiration in mitochondria, for the treatment of cancerous cells. But due to the subcellular level, these approaches were significantly less effective. Nowadays more research is focused on mitochondrial targeting by developing formulations to obtain effective access of the mitochondria. One scientist developed an iron oxide nanoparticle for the mitochondria targeted PTT (Photo-thermal therapy), the iron oxide NPs coumarin functionalized found to be primarily accumulated in endoplasmic reticulum. They demonstrated higher *in vivo* efficacy of the formulation (Jung et al., 2017).

### Factors Affecting the Intracellular Targeted Drug Delivery Systems

There are various factors that affect intracellular targeted drug delivery, such as size, shape, hydrophobicity, surface charge, and surface modification (Qiu et al., 2010; Foroozandeh and Aziz, 2018). The endocytic uptake of NPs is mostly affected by the above mentioned parameters. In this section a brief explanation has been provided on some of the factors that govern uptake and intracellular targeting of drugs.

#### Size and Shape of NPs

**Figure 2:**
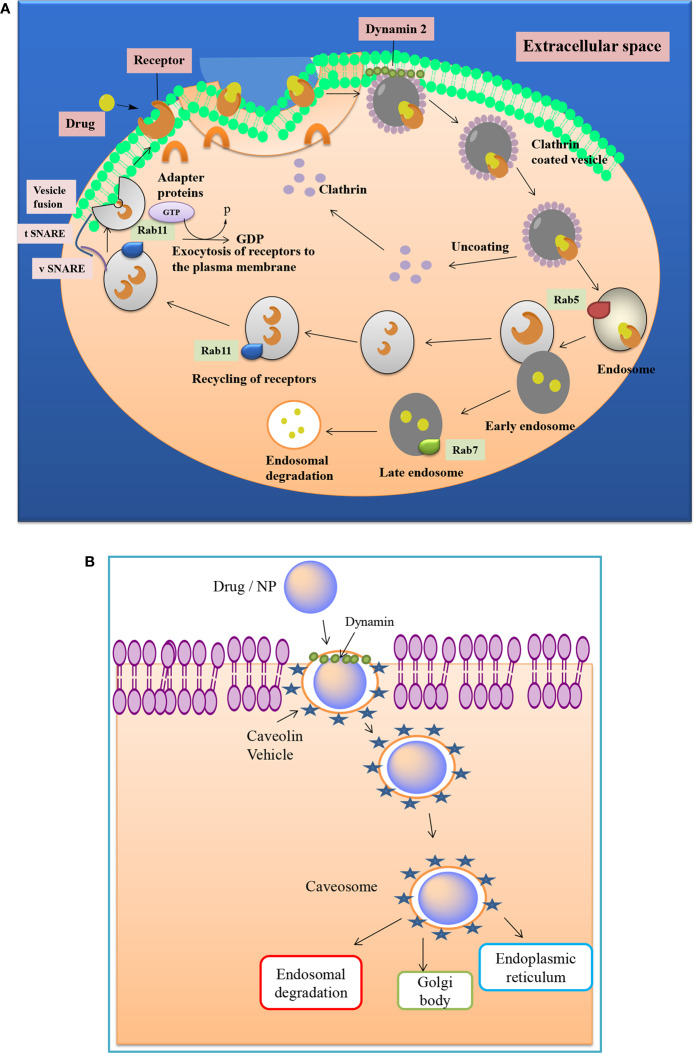
**(A)** Clathrin mediated drug transport across the cell membrane. Cell surface receptors bind the exogenous ligand and internalize the moiety by formation of clathrin, which implicates the role of various proteins like Rab, GTPases, and SNARE proteins etc. **(B)** Caveolae mediated transport of a drug or nanoparticle. It involves the formation of bulb-shaped, 50-60 nm plasma membrane invaginations, called caveolae, whose formation is driven by integral membrane proteins and peripheral membrane proteins.

Effective NPs uptake is highly dependent on the size and shape of the Nanomaterials (Zhu et al., 2013). It also governs the cytotoxic potential of nanomaterials (Nel et al., 2009). The optimum size of NPs for efficient uptake into the cell membrane is 50 nm (Chou et al., 2011). NPs with a size ranging from 120-150 nm primarily get internalized through caveolin and clathrin mediated endocytosis pathways (**Figures 2A, B**). Direct entry of NPs into the bilayer of cells can be enhanced by reducing the size of NPs (Zhu et al., 2013). Along with size, the shape of NP also plays a vital role in the absorption and trafficking of molecules through various compartments. Researchers have observed the consequences of shape of AuNP on uptake into HeLa cells and found that the spherical shaped AuNPs shows five times higher uptake than rod shaped AuNPs. Filomicelles have a role in transport and trafficking, and it has been found that they have a greater circulation half-life than a spherically shaped NP (Foroozandeh and Aziz, 2018).

#### Surface Charge

Surface charge is the most crucial factor which readily determines the cellular uptake of nanoparticles. The surface charge can be determined by Zeta potential measurement and can be positive or negative. The highly negatively charged cell membrane enhances the uptake of the positive charge nanoparticles. The higher uptake of the positive charge particles may create distortion of the membrane integrity and this can lead to an increase in cellular toxicity. The uptake of AuNPs *via* different pathways depends on the surface charge. The positively charge AuNPs can be internalized *via* micropinocytosis and clathrin mediated endocytosis (Panariti et al., 2012).

#### Surface Modification

The surface modification can be of great potential in increasing the uptake of NP into the cell *via* various trafficking pathways; and can be helpful in reducing the toxicity of NPs, governing the stability and their fate. The uptake can be increased by increasing the positive charge on NPs *via* surface modification, adding an amino (-NH_2_) group on the surface. The negative charge can be enhanced *via* carboxylation (-COOH) which increases the cellular uptake (Alexis et al., 2008).

## Significance of Subcellular Drug Targeting

The distribution of drugs and its subsequent access to the targeted location is the main aim of drug targeting. More often than not, the target location plays a very crucial role in drug delivery, as sometimes the target location is inside the cells, or more precisely, within cellular organelles. The significance of subcellular targeting is expected to improve potency and to minimize related side effects. There are some properties which significantly influence targeting and subsequent organ accumulation of molecules viz. surrounding pH, resident molecules itself, electronic potential, lipid-bilayer composition, and membrane bound proteins (Duvvuri et al., 2004). Nowadays, pharmaceutical nanotechnology can overlook the limitations of conventional administration and poor bioavailability of drug molecules, and by using new and improved nanoformulations, which include different kinds of carriers and conjugates, overall improvement in the pharmacokinetics and pharmacodynamics behavior has been observed, facilitating the implementation of novel DDS in clinical use.

Organ targeting by transforming a drug molecule into polymer coated microspheres improves the pharmacokinetic parameters for local pulmonary therapy, minimizing drug accumulation in the heart, followed by minimizing cardiotoxicity. Incorporating polyethylene glycol (PEG) into nanoformulations overcomes the first-pass elimination from blood and bypasses the reticuloendothelial (RES) system. PEGylated liposomes of doxorubicin presented modified pharmacokinetic data as compared to plain doxorubicin. Targeting molecules into subcellular locations significantly improves the bioavailability of drugs. Internalization of drug molecules depends on the polymer-ligand interaction. The main aim of a targeted drug delivery system is to control biodistribution and to improve pharmacokinetics of the drug by controlling physiochemical and biochemical properties (Leucuta, 2014).

## Role of Carriers in Drug Targeting

Although great efforts have been practiced in targeting cell surface receptors, very limited therapeutic advantages have been availed. The recent focus has since, therefore, shifted to strategies of targeting subcellular components to achieve specificity and maximum therapeutic benefits (Rajendran et al., 2010; Leucuta, 2014). The maximum therapeutic efficacy of a drug (either macromolecular or DNA, protein and peptide based drugs, oligonucleotides, siRNA etc.) can be exploited by maneuvering these with the help of some specialized carriers to various subcellular compartments like nuclear and mitochondrial targeting, to achieve safe and efficacious carriage to the desired sites of actions (Lamade et al., 2019; Nurunnabi et al., 2019; Sun et al., 2019; Wang D. et al., 2019). In this context a number of carriers have been designed, sometimes to exploit either a particular subcellular component or a drug delivery process (Huang et al., 2016). Various carriers have been formulated for the effective delivery of drug molecules across a wide spectrum of subcellular components which regulate the drug's subcellular pharmacokinetics and pharmacodynamics (**Table 2**) (Paillard et al., 2010; Leucuta, 2014; Sun et al., 2019).

**Table 2:** Types of subcellular targeted drug delivery systems.

S.No.	Target	Drug/Formulation	Mechanism	Disease	Ref.
1.	Plasma membrane	Enfuvirtide C34	Membrane targeted inhibition of HIV fusion complex	AIDS-HIV	(Rajendran et al., 2010)
2.	Nuclear targeting	RGD peptide systems	regression in αvβ3-overexpressing cancer	Human breast cancer and melanoma	(Park et al., 2016; Nurunnabi et al., 2019)
3.	Nuclear targeting	Cell penetrating TAT peptides	enhancing cytoplasmic delivery, transfection efficiency	Liver cancer	(Richard et al., 2003; Song et al., 2010; Nurunnabi et al., 2019)
4.	Nuclear targeting	Viral like particles/liposomes	Targeted gene delivery by viral-mediated vectors	Carcinoma	(Nayerossadat et al., 2012; Leucuta, 2014; Sung and Kim, 2019)
5.	Endosomes (early)	Cholesterol conjugated β-secretase inhibitors	Membrane attachment followed by endocytosis into endosomes	Alzheimer's disease	(Cheng et al., 2007; Cole and Vassar, 2007)
6.	Mitochondria	Paclitaxel liposomes	Better cellular uptake and accumulation in mitochondria and enhanced cytotoxicity	Liver cancer	(Biswas et al., 2011; Wang et al., 2017)
7.	Mitochondria	Curcumin DQAsomes	Appreciable antioxidant and anti-inflammatory efficacy	Acute lung injury	(Zupančič et al., 2014)
8.	Mitochondria	Doxorubicin Cerasomes	greater drug accumulation in mitochondria and greater antitumor effect	Targeted Tumor therapy	(Wang et al., 2015)
9.	Mitochondria	Coenzyme Q10 Micelles	Enhanced cellular uptake and higher mitochondrial accumulation	oxidative stress and inflammation	(Sharma et al., 2012)
10.	Mitochondria	α-tocopheryl succinate Mesoporous Silica NPs	Intracellular uptake and mitochondrial accumulation	Anticancer therapy	(Qu et al., 2016)
11.	Golgi Bodies and ER	Conjugated Antigenic peptide	Targeted presentation on MHC class I complex by conjugation to STX-B	Ovarian cancer, intestinal cancer and lymphomas	(Morse et al., 2011; Reeves and James, 2017)
12.	Golgi Bodies and ER	Rhodamine-loaded PLGA (polylactic-co-glycolic acid) NPs	colocalization with early endosomes, late endosomes, lysosomes, endoplasmic reticulum (ER), and Golgi apparatus	Respiratory, gut and renal targeted delivery	(Cartiera et al., 2009)
13.	Cell membrane (receptors)	Transferrin eight arm polyethylene glycol-dihydroartemisinine nanoparticles	Transferrin receptors mediated cellular internalization	Lewis Lung carcinoma	(Liu et al., 2016; Liu et al., 2017)
14.	Cell membrane (receptors)	Adenosine conjugated Solid Lipid Nanoparticles	G-protein coupled receptors mediated cellular internalization	Human cancer	(Swami et al., 2015; Ma et al., 2018)
15.	Epidermal Growth Factor Receptors	GE11 peptide conjugated Exosomes for microRNA	efficient delivery of microRNA (miRNA) to epidermal growth factor receptor (EGFR)-expressing breast cancer cells	Breast cancer	(Li et al., 2005; Ohno et al., 2013)

**Figure 3:**
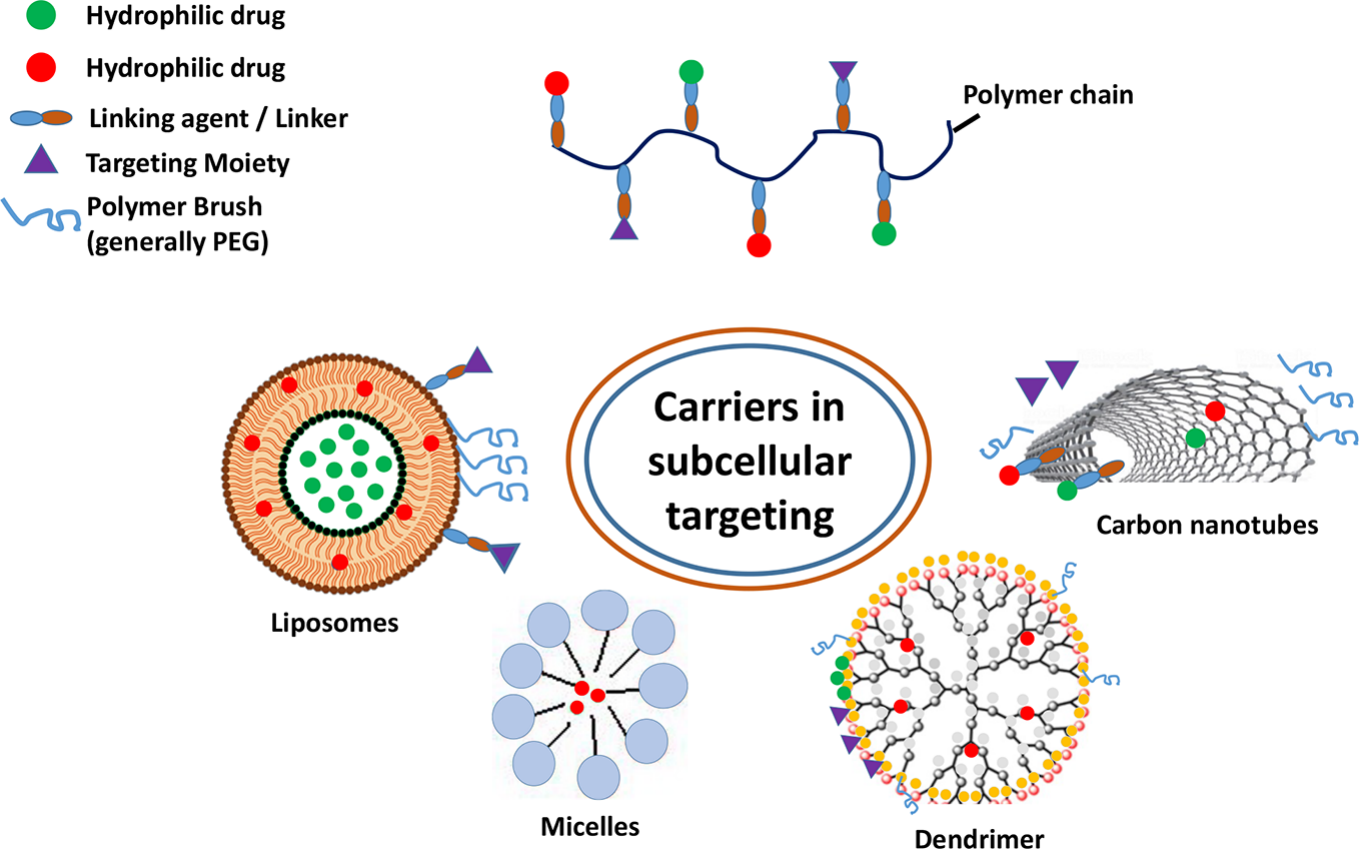
Various carriers and their components employed in subcellular targeting. The nanocarriers are composed of hydrophobic and/or hydrophilic polymeric chains capable of encapsulating and delivering both kinds of therapeutic payloads.

In general, carriers have been conceptualized with the purpose of delivering drugs by targeting nuclear components such that these molecules directly regulate gene expression or protein transcription processes (**Figure 3**) (Ahmad et al., 2019). In other instances, a specific subcellular component, such as early or late endosomes, help drug carriers in one way or the other e.g. in pH mediated drug release, exocytosis, and subcellular drug distribution etc. (Bae et al., 2005; Yuanjie Zhu and Yuanjie Zhu, 2019). A significant example is the designing library of mitochondrial targeting tetra-peptides employed in targeting mitochondrial dysfunction in neurological and cardiovascular disorders. These molecules exhibit limited toxicity profiles with significantly higher efficacies in providing protection against exogenously-induced damages. Along with these characters, targeting agents possessed enhanced colocalization with mitochondria and regulated subcellular accumulation and eminent capacity as a targeted delivery vehicle (Sun et al., 2019).

**Figure 4:**
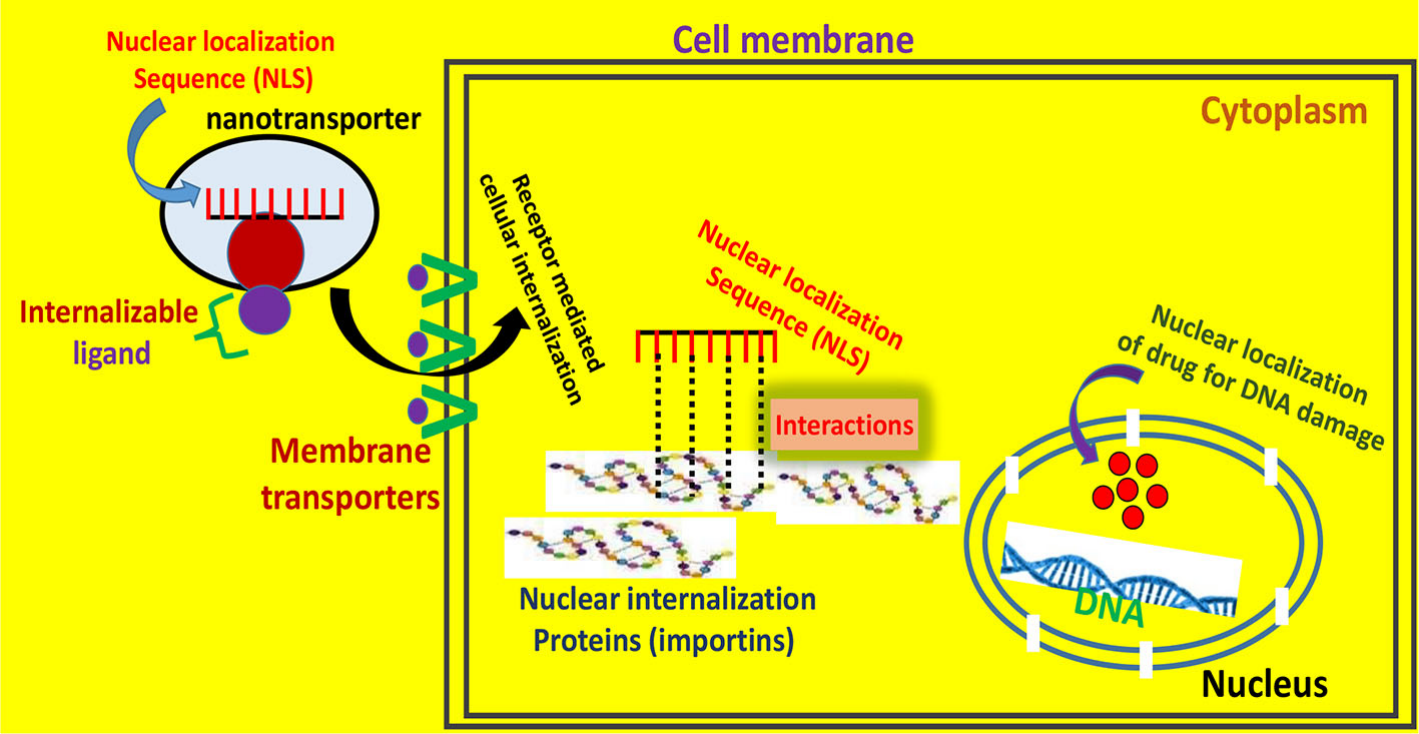
Membrane and subcellular trafficking implicated in the nuclear localization mechanism of drugs. The nanotransporter comprises an internalizable ligand which facilitates the receptor mediated cell entry, and a nuclear targeting moiety (nuclear localization sequence; NLS) which enters the nuclear region and regulates the gene expression.

Other studies include the designing of mitochondria targeted mesoporous silica nanoparticles containing the hydrophobic anticancer agent α-Tocopheryl Succinate. An effective mitochondria targeting ligand, triphenylphosphonium (TPP), was employed for surface functionalization. After conducting various experiments the authors concluded that the nanocarriers imparted higher efficacy to drugs indicating a promising approach of targeting the mitochondria (Qu et al., 2016). In another approach, multifunctional nanocarriers were designed for the selective delivery of coenzyme Q10 to mitochondria and the efficacy of nanocarriers was assessed in terms of two different experimental paradigms viz. oxidative stress and inflammation. The authors suggested that the nanocarriers were effective and preserved and protected the drug effectiveness during its delivery (Sharma et al., 2012). Likewise, other nanocarrier mediated mitochondrial targeting paradigms like curcumin DQAsomes, rhodamine-123-conjugated polymeric liposomes were also designed for different aims. Curcumin loaded DQAsomes potentiated the antioxidant efficacy of curcumin (Zupančič et al., 2014), while modified liposomes exhibited better cellular uptake by HeLa cells. A high degree of accumulation of these rhodamine-123-conjugated polymeric liposomes could also be observed in mitochondria. In terms of cytotoxic efficacy, appreciably enhanced cell cytotoxicity could be observed with modified liposomes as compared to non-targeted liposomes (Biswas et al., 2011). Along with the mitochondrial targeting, nuclear targeting strategies such as RGD peptide systems, cell penetrating TAT peptides etc. have been introduced, which can carry and deliver the therapeutics and exhibit high promises in nuclear targeted drug delivery paradigms (Nurunnabi et al., 2019). In a recent study, a RGD peptide was used in combination with other peptides and a nuclear localization sequence, specifically for nuclear targeting, where researchers delivered a precise transportation of a tracing reagent to the nucleus. A multifunctional fluorescent probe was combined with the RGD peptide and the nuclear localization sequence, after binding to cell surface receptors, effectively being internalized into the nucleus (Cheng et al., 2017). In another concept, Yang et al. functionalized nanoparticles with the RGD peptide to enhance cellular uptake with an aim to enhance therapeutic efficacy of nanoparticles through their effective targeting into the nucleus. These peptide coated gold NPs exhibited five-fold enhancement in the nanoparticle uptake and effective nuclear localization. This effective cellular uptake and nuclear localization imparted more therapeutic efficiency to gold NPs in their anti-cancer approach (**Figure 4**) (Yang et al., 2014). Colin et al. report that a major barrier for efficient gene therapy is gene transit across the nuclear envelope. The RGD peptide is shown to mediate the enhanced internalization of the plasmid and synergistic gene expression. This enhanced internalization is due to increased nuclear transfer, which is an energy dependent mechanism. The authors concluded that the oligolysine-RGD peptide acts as a nuclear localization signal and RGD aids it by binding to RGD and recognizing integrin proteins (Colin et al., 2001). Along with all these approaches, the RGD peptide has extensively been used with other nanomedicine based platforms viz. RGD mediated delivery of quantum dots for tumor cell imaging (Cai et al., 2006; Lieleg et al., 2007; Smith et al., 2008), iron-oxide NPs with RGD peptide for endothelial cell labeling and imaging (Montet et al., 2006; van Tilborg et al., 2008; Almutairi et al., 2009; Schottelius et al., 2009; Sugahara et al., 2009), and RGD conjugated dendrimers for angiogenesis imaging etc. (Almutairi et al., 2009).

Apart from these, certain plasma membrane targeting approaches have also been designed, which include drug Enfuvirtide-C34 against HIV-AIDS, where the lipid linkage enhances the membrane targeting and better inhibition of HIV fusion complex, Myr-proS1 for HBV fusion inhibition by targeting the HBV fusion complex, and Pepducin as GPCR modulator for an effective modulation of the GPCR signaling (Rajendran et al., 2010). Apart from nuclear, mitochondria, and plasma membrane targeting, Golgi apparatus and endoplasmic reticulum have also been the targets of drug delivery approaches. Biodegradable Rhodamine-loaded PLGA (polylactic-co-glycolic acid) NPs were assessed in epithelial cells to demonstrate colocalization with specific organelles: early endosomes, late endosomes, lysosomes, endoplasmic reticulum (ER), and Golgi apparatus (Cartiera et al., 2009). This platform was employed for respiratory, gut, and renal targeted delivery. Another system for targeting endoplasmic reticulum and Golgi apparatus was composed of conjugated Antigenic peptide directed against ovarian cancers, intestinal cancers, and lymphomas by targeting the presentation on the MHC class I complex. These approaches offered the advantage of considerably enhanced cellular uptake, colocalization with the organelles of interest, and offering biocompatible and relatively tolerable subcellular targeting delivery ssytems (Morse et al., 2011).

In the context of receptor targeting, discovery of human epidermal growth factor receptor 2 (HER2) has revolutionized anti-cancer therapies, in particular for personalized medicine in HER2 receptor amplified breast cancer patients. Recent research on HER2 receptor targeted anti-HER2 drug conjugate (HER2-ADC) has become the pillars of treatment against advanced HER2 positive breast cancers. These are composed of antibodies against the extracellular domain of human HER2 receptors and contain the monoclonal antibody (e.g. trastuzumab) that binds to HER2 and fuses with a cleavable linkage to another cytotoxic anti-microtubule reagent (e.g. tubulysin) with very potent anti-neoplastic antibodies. Upon binding to the target receptors HER2, antibody drug conjugate is internalized into cancer cells and is translocated to lysosomes where the linker is cleaved. The drug is then delivered to HER2 expressing cancer cells and finally blocks the cancer cell division, resulting in G2/M phase arresting, apoptosis, and reduced proliferation of HER2 positive cancer (Next-Generation Anti–HER2-Antibody Drug Conjugates, ; Rinnerthaler et al., 2019; D'Amico et al., 2019).

Targeting transferrin receptors in the brain, for the delivery of a drug payload across the blood brain barrier (BBB), has always been a considerable challenge for researchers working on central nervous system diseases. Although antibodies have been employed against transferrin receptors, nothing could be achieved by the mere use of naïve antibodies. Research on the engineering of those antibodies against transferrin receptors then started and various approaches were undertaken. One concept describes the conjugation of mouse antibody to another anti-amyloid plaque antibody for targeting the brain side of the BBB (Niewoehner et al., 2014). Another strategy suggests that engineering of anti-transferrin antibodies, to reduce their affinity against transferrin receptors, lead to the significant enhancement of their accumulation in the brain parenchyma (Paterson and Webster, 2016).

At molecular levels, few specialized nanoparticulate drug delivery nanocarriers have evolved that can safely and effectively carry drugs for nuclear targeting in such a way that the subcellular internalization can surpass the biological blockades either passively or *via* active targeting (e.g. receptor mediated drug delivery). These approaches of intracellular drug distribution with specialized drug delivery systems is expected to be clinically beneficial and will be a great boon for pharmaceutical nanotechnological platforms (Leucuta, 2014). Different criteria have been set for the drug carriers with subcellular targeting purposes, e.g. it should be inert, which means the drug should be released in its active form after reaching subcellular drug targets, the drug and carrier linkage should be strong and stable, it must get through the biological barrier points, and should be non-toxic and non-immunogenic etc (Schneider et al., 1984). Sometimes the drug carrier geometry also influences its uptake or endocytosis and ultimately the subcellular targeting, lysosomal transport, its residency in the prelysosomal compartments, the half-life of the drug in circulation, as well its specificity etc. (Muro et al., 2008). The capacity of a medicinally active compound to bind to its specific target in the cell is intimately associated with its potential to target the molecular events inside the cell organelles. However, often the compound itself does not have the potential to permeate inside the cell and therefore requires the aid of a carrier molecule, sometimes to attain metabolic stability, to attain maximal therapeutic efficacy, to control subcellular disposition, or to achieve organelle oriented targeting effects (D'Souza and Weissig, 2009). Different internalization mechanisms have also been proposed for the carriers directed to the subcellular targets viz. phagocytosis, pinocytosis, clathrin mediated endocytosis, caveolae mediated endocytosis, *via* formation of early and late endosomes, and macropinocytosis etc (Guo et al., 2020).

## Endosomal Escape of Drugs and Carrier Systems for Subcellular Targeting

In spite of all the benefits of targeting drugs to cellular and subcellular locations, the targeting carriers may undergo entrapment followed by degradation in the acidic chambers of endosomal or lysosomal pathways. Optimization measures have been carried out to escape the endosomes and a greater understanding of measures that govern endosomal escape is required, as this is the most crucial and rate determining step in the delivery of therapeutics (Smith et al., 2019). These measures include incorporation of functional groups with positive charge, pH sensitive moieties, utilization cationic polymers and lipids e.g. poly (ethylene imine), endosomal swelling strategies, and disrupting the endosomal membrane etc. Recent approaches also undertake the use of bacterial toxins, e.g. purified Listeriolysin O (LLO) toxin, as a medium to promote escape from the degrading environment of lysosomes and to avoid the breakdown and degradation of the delivery system through the endosomal lysosomal network in the cell (Plaza-GA et al., 2019). However all of these strategies are associated with their respective limitations and designing a carrier system which can achieve a 100% endosomal escape is still a far flung achievement (Cupic et al., 2018).

Some recent studies, in the context of endosomal escape, include the formulation of a drug carrier made up of albumin combined with palmitoyl-cyclic-(D-Arg)_12_. This carrier was used as endosome mimicking liposomes and data further suggested that palmitoyl-cyclic-(D-Arg)_12_/HAS is internalized inside the cell through induction of micropinocytosis, and exhibits endosomal escape (Ichimizu et al., 2019). Another strategy describes conjugation of stromal-derived factor 1α (SN_21_) with membrane lytic peptides which induces micropinocytosis or membrane lysis and aids in the successful delivery of functional siRNAs and proteins. It also gives the loaded drug cargo the ability to be released from endosomes or endosomal escape (Arafiles et al., 2020). Fraire et al., describe the escape from endosomes in terms of endosomal rupture through the photothermal properties of gold nanoparticles by vapor bubble formation and heat induction. In this approach the loaded cargo of siRNA is released intact and with equal efficacy, and heat mediated endosomal escape is dependent on the number of gold nanoparticles per endosome. This approach also had the advantage of not disturbing the cellular homeostasis (Fraire et al., 2020).

To overcome the major challenge of endosomal sequestration of RNAs therapeutics Orellana et al., exploited the difference in solute concentration between nascent endosomes and cytoplasm which resulted in osmotic swelling and the bursting of endosomes. They employed the nigericin molecule to exchange potassium and hydrogen ions and enabled the folate-RNA conjugates to escape from endosomal entrapment or capture (Orellana et al., 2019). Some other recently described approaches for endosomal escape include, but are not limited to, the formulation of pH buffering particles which build up the osmotic pressure and exhibit osmolytic endosomal escape - membrane disrupting particles which destabilize the membrane and gelatinous nanoparticles which exhibit endosomal escape *via* swelling and endosomal rupturing mechanisms (Cupic et al., 2018). Especially for charge containing drugs and carriers, the incorporation of a charged molecule inside the membrane which leads to the breakdown of membrane symmetry, creating alterations and disturbances in the electrostatic interactions, alterations of the protonation levels of pH responsive drug delivery systems, charge mediated changes in the membrane tension and consequent membrane ruptures, and ion mediated changes in the osmotic pressure inside endosomes are some of the recently studied mechanisms for endosomal escape (Tian and Ma, 2012; Lönn et al., 2016; Allen et al., 2018).

## Current Scenario of Subcellular Drug Targeting

With the advent of serious and toxic side effects associated with current conventional drugs with generalized distribution and non-specific targeted therapies, researchers have directed their focus on developing specific and subcellular targeted therapies so as to maximally enhance therapeutic benefits and to minimize off-target effects. Recently wide spectrums of subcellular targeting strategies have come into practice, which can dispose drugs at therapeutic targets and organelles (e.g. lysosomes, mitochondria and nucleus etc.) *via* multiple mechanisms (Chakraborty et al., 2018; Wang F. et al., 2019; Yuanjie Zhu and Yuanjie Zhu, 2019; Guo et al., 2020).

Recently, researchers formulated a reaction system comprised of copper catalyzed azide-alkyne cycloaddition to specifically target the preferential accumulation of the resveratrol derived drug in the mitochondria of living cells. The system possessed high catalytic efficiency and exhibited focused drug synthesis through site-directed specificity. The reaction system developed various advantages such as the avoidance of the troublesome delivery process and non-specific distribution of obnoxious moieties, possibilities of multi-drug synthesis, and high biocompatibility etc. (Wang F. et al., 2019). Similarly, another functionalized colloidal nanoparticulate system with chemical and biochemical surface functionalization was developed, which took into account the recent concept of nanoprobes. Polyacrylate material was employed to form colloidal nanoprobes with the specific aim of targeting subcellular compartments such that the nanobioconjugates can facilitate nanoparticle-cell interaction, helping the endocytosis lead to localized subcellular disposition of the active molecule (Chakraborty et al., 2018).

Another functional subcellular targeting strategy describes the DNA origami based nanostructures for targeting late endosomal and lysosomal drug distribution in subcellular localities. Fluorescent triangular structures were designed which were safe, biocompatible, efficient, and evaluated in U87MG cells. These nanostructures were modified with Cy5 dye, targeted against endosomes and Golgi apparatus and showed respective efficient colocalization under a confocal laser scanning microscope (Yuanjie Zhu and Yuanjie Zhu, 2019).

Recently, Kang et al., developed a mitochondrial targeting strategy by bypassing the multi-drug resistance, by formulating liposomes which carried resveratrol and targeted mitochondria. These were aimed at providing enhanced circulation of drugs in the blood and exhibited an enhanced retention and permeability (EPR) effect. The liposomes were 120 nm and possessed positive zeta potential, and when loaded with rhodamine, showed effective uptake in the cells under confocal microscope. The enhanced uptake was also demonstrated by LCMS/MS quantified resveratrol in the subcellular organelles. The mitochondrial membrane dissipation, ROS production, and cell cytotoxicity results demonstrated that these liposomes can provide a vital subcellular targeting strategy in the cancer treatment (Kang and Ko, 2019).

When the administered drug meets various biological and physiological barriers, only a fraction of drugs reach the target site and exhibits a pharmacological response, while the majority is disposed to off target sites and leads to undesired and harmful effects. To overcome these limitations another strategy suggests the design of cleavable nitrogen-terminal extensions in drug carriers such that it allows the subcellular translocation of nuclear targeting compounds such as TAT or RGD peptides into the nucleus. The desired drug can also be introduced along with these nuclear targeting proteins or peptides which exhibit a significant benefit in nuclear targeting drug delivery practices (Nurunnabi et al., 2019).

Babikova et al., recently developed a multi-functional triblock copolymer for mitochondrial targeting application which comprised of a saccharide attached polyoxyethylene block, an amphiphilic diblock copolymer, and a biodegradable hydrophobic component. The polymeric carriers were loaded with anti-cancer drugs of plant origin i.e. curcumin. The mitochondrial targeting ability was assessed in terms of the cell cytotoxicity, apoptosis potential, and NF-κB inhibition potential and all of these parameters were found to be superior in the polymeric carriers than in the naïve drug alone or in simple unconjugated carriers (Babikova et al., 2019). Another recent mitochondrial targeting strategy takes into account the enhanced temperatures of mitochondria to develop a finely tuned thermoresponsive drug carrier for paclitaxel, an anti-cancer drug. The carrier comprised of polymeric components like polyacrylamide and ply(N-isopropylacrylamide) and in all the *in vitro* experimentation results established the superior efficacy at higher temperatures. The nanocarriers exhibited superior efficacy in terms of cell cytotoxicity, colocalization, and cellular uptake of drug loaded nanocarriers over the naïve drug alone (Wang D. et al., 2019).

It is known that photocaging helps in the accurately site specific and non-invading release of drug molecules inside the cell, however, subcellular organelles in the cells are placed in such a way that precise targeting of these with light becomes non-workable. Henceforth, organ specific photocages can facilitate the release of these pharmaceutical compounds in subcellular localities. Kand et al., chemically functionalized and conjugated the BODIPY based photocages and their derivatives for the specific targeting of lysosomes, endoplasmic reticulum, and mitochondria. They also demonstrated that visible light could cause the selective photorelease of 2,4 DNPH, a mitochondrial uncoupler and puromycin, a protein synthesis inhibitor. These photocages also provide enhanced efficacy to pharmacological compounds because of the localized and precipitous delivery (Kand et al., 2019).

Ender et al. report that certain subcellular vesicles produced by the cancerous cell arbitrate cancer resistance against anti-cancer drug molecules through different mechanisms. These vesicles are either exosomes or ectosomes, as categorized depending on their size, shape, content, and mechanism of production. The recent advances in subcellular targeted therapies against these vesicles and an in-depth knowledge about the mechanism of this subcellular targeting strategy will be a great boon in the fight against cancer resistance (Ender et al., 2019).

## Conclusion and Future Perspective

In this review we have summarized some aspects of membrane trafficking and subcellular targeting phenomena. How membrane trafficking and its various aspects and components help in the regulation of drug targeting have been briefly described. Different sub-components of drug targeting schemes have been shortly touched on and how the optimization of different drug related parameters can aid in the formulation of subcellular targeting approaches is also discussed. Lastly, how various molecules and particulate carriers aid in the drug delivery to subcellular targets has been briefly discussed. Once pharmacologically active biomolecules are delivered to the organs, tissues, and cell vicinities is accomplished, the next big task to achieve is the drug delivery inside the cells and to the specific subcellular organelles. A great deal of achievements have been fulfilled recently as far as the targeted delivery of bioactives to the subcellular compartments is concerned. Researchers have carried out exhaustive evaluation of diverse and versatile physicochemical and biological parameters for the targeted delivery of drugs to the subcellular localizations. Many techniques and processes have been standardized and established with regards to subcellular drug targeting so as to obtain the maximal therapeutic efficacy and to minimize the off-target effects, unwanted, and adverse drug reactions. These approaches will be greatly helpful in the development of personalized and precision medicine. Among the entire subcellular targeting paradigm, nuclear, mitochondrial, and lysosomal targeting strategies have achieved great accomplishments and have provided great advantages. Still, some lacunae remain and will be filled to maximally exploit subcellular targeting strategies. A lot of work remains to be done as far as the prediction of subcellular drug localization is concerned. The use of multi-labelled proteins in this context has increased significantly but has not been tapped to its maximum potential. The development of drugs with multiple subcellular targeting possibilities is still in its initial stages. An in depth understanding of drug internalization, accumulation, biotransformation, and excretion mechanisms is also needed. We hope this review will be of great help to researchers working in the field of targeted drug delivery and personalized medicine.

## Author Contributions

AK, AA, AV, and RK have written the manuscript and prepared figures and tables. RK has finally edited the manuscript.

## Conflict of Interest

The authors declare that the research was conducted in the absence of any commercial or financial relationships that could be construed as a potential conflict of interest.
